# Vertebral scale system to measure heart size in thoracic radiographs of Indian Spitz, Labrador retriever and Mongrel dogs

**DOI:** 10.14202/vetworld.2016.371-376

**Published:** 2016-04-14

**Authors:** Deepti Bodh, Mozammel Hoque, Abhishek Chandra Saxena, Mudasir Bashir Gugjoo, Deepika Bist, J. K. Chaudhary

**Affiliations:** 1 Division of Surgery, Indian Veterinary Research Institute, Izatnagar, Uttar Pradesh, India; 2 Division of Livestock Economics, Statistics and Information Technology, Indian Veterinary Research Institute, Izatnagar, Uttar Pradesh, India

**Keywords:** dogs, radiography, recumbency, thorax, vertebral heart scale

## Abstract

**Aim:**

To establish reference values of vertebral heart score (VHS) in Indian Spitz, Labrador retriever, and Mongrel dogs; to assess applicability of VHS in these three dog breeds; to determine if breed, recumbency side, gender, body weight, and thoracic depth (TD) to thoracic width (TW) ratio has an influence on the VHS measurement in these dog breeds.

**Materials and Methods:**

A total of 60, client owned, clinically healthy Indian Spitz (n=20, mean age = 4.25±2.15 years, body weight = 11.87±2.7 kg), Labrador retriever (n=20, mean age = 4.75±1.91 years, body weight = 27.31±5.43 kg), and Mongrel dogs (n=20, mean age = 4.25±1.52 years, body weight = 16.25±3.99 kg), having no radiological and clinical signs of cardiovascular or pulmonary disease were included in the study. All dogs were restrained manually and left lateral (LL) and right lateral (RL) radiographic views were obtained. The size of heart in lateral radiographs was calculated using VHS method. Besides, the TD, TW and TD: TW were calculated to determine the type of thoracic conformation in the dog breeds. In addition to this, the effect of breed, side of recumbency, gender, body weight, and TD to TW ratio on the calculation of VHS was determined.

**Results:**

VHS was calculated in all the animals of the breeds. VHS in Spitz and Labrador retriever was significantly (p<0.0001, p<0.0001, respectively) >9.7±0.5 v. RL and LL VHS in Mongrel dog was significantly (p<0.037) >9.7±0.5 v. Significant (p<0.05) differences in the VHS were observed among Spitz, Labrador retriever and Mongrel dogs, being higher for Labrador retriever followed by Spitz and Mongrel dogs. VHS in RL recumbency was significantly (p<0.001) greater than VHS in LL recumbency in all three breeds. LL and RL VHS correlated significantly with each other in Spitz (r=0.58; p=0.02), Labrador retriever (r=0.87; p<0.0001), and Mongrel dogs (r=0.93; p<0.0001). Significant (p<0.05) differences in the TD and TW were observed among Spitz, Labrador retriever, and Mongrel dogs. Non-significant effect of gender, body weight, and TD to TW ratio on the VHS measurement was observed in each dog breed.

**Conclusion:**

Breed-specific VHS reference ranges should be used for the objective measurement of heart size in dogs. Furthermore, the radiographic view should also be taken into consideration to avoid any erroneous interpretation of cardiac enlargement in dogs.

## Introduction

Despite the advent of echocardiography, thoracic radiography remains an essential part of the diagnosis and management of cardiac disease in dogs. Alteration in the shape and size of cardiac silhouette, abnormal size, shape of pulmonary vessels and the presence of pulmonary edema or ascitis on thoracic radiographs are often the hallmarks for radiographic diagnosis of cardiac diseases in dogs [1].

For evaluation of cardiac silhouette and to maximize the accuracy of radiographic diagnosis of cardiac disease in dogs, a variety of subjective as well as objective methods were proposed [2,3]. These methods were found unsuitable in clinical practice owing to marked interbreed and individual variations in the axis of the heart and its silhouette, thoracic conformation, respiratory phase, rib superimposition, and imprecise measurement points [4-7].

To overcome these limitations, the vertebral heart scale (VHS) method was developed which involved measuring the long axis (LA) and short axis (SA) dimensions of heart in the lateral radiograph and comparing their sum to the mid thoracic vertebral bodies starting from the anterior edge of the 4^th^ thoracic vertebrae. A mean VHS of 9.7±0.5 v obtained from lateral radiograph of 100 clinically normal adult dogs of different breeds was considered as a clinically useful upper limit for normal heart size in dogs [8].

The universal VHS of 9.7±0.5 v could not be applied to all the dogs as values wider or higher than this have been reported in different dog breeds [9,10]. Furthermore, the effect of breed, recumbency, gender, body weight, and thoracic depth (TD) to thoracic width (TW) ratio on computation of VHS was unknown initially. However, recent studies have reported a significant influence of breed [4,6,7,9,10], recumbency side [4,6,11], gender [12], and body weight [13] on the VHS in dogs.

Considering aforementioned reports, this study was designed to establish and compare reference VHS values in Indian Spitz, Labrador retriever, and Mongrel dogs; to assess the applicability of normal VHS value proposed by Buchanan and Bucheler in these dog breeds; to determine if there is any effect of breed, side of recumbency, gender, body weight, and TD to TW ratio on the VHS measurement in such dog breeds.

## Materials and Methods

### Ethical approval

This study being a part of larger study for doctorate thesis, informed consent had been obtained from all the clients before start of any examination procedure. The radiographic examinations were carried out as per the standard procedure without harming the animals.

### Dogs

A total of 60, client owned, clinically healthy, adult Spitz (n=20; mean age = 4.25±2.15 years; mean body weight = 11.87±2.7 kg), Labrador retriever (n=20; mean age = 4.75±1.91 years; mean body weight = 27.31±5.43 kg), and Mongrel dogs (n=20; mean age = 4.25±1.52 years; mean body weight = 16.25±3.99 kg) were made subject of the study. All dogs presented for general health check up to the Referral Veterinary Polyclinics, Indian Veterinary Research Institute were subjected to thorough clinical, radiographic, electrocardiographic, echocardiographic, and complete hemato-biochemical examination. Only those considered healthy and free from any cardiovascular disease underwent a radiographic examination of the chest.

### Radiographic examination

**Figure 1:**
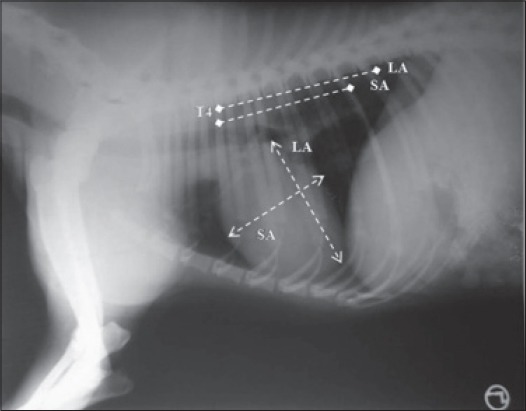
Long axis (LA) and short axis (SA) measurement of heart in lateral recumbency for calculation of VHS. T4 is the fourth thoracic vertebra.

The radiographic examination included right lateral (RL) and left lateral (LL) views. Radiography was performed without sedation using standard exposure techniques. All radiographs were taken at the time of full inspiration. An attempt was made to keep the chest of animal as close to the film as possible, to include all the thoracic vertebrae in radiographs and to avoid any rotation of the body of animal. The radiographs were evaluated qualitatively to exclude animals that presented any radiographic change. Rotated, oblique or expiratory radiographs were excluded from the study. Quantitative evaluation of the radiographs was performed by measuring vertebral heart score (VHS) using the method described by Buchanan and Bucheler [8]. In lateral thoracic radiographs, LA of heart was measured from the ventral border of the largest main stem bronchus seen in cross section to the most distant ventral contour of the cardiac apex using an adjustable caliper (Figure-1). The caliper was then repositioned over the thoracic vertebrae beginning with the cranial edge of the fouth thoracic vertebrae (T_4_). Distance spanned by the caliper was estimated to the nearest 0.1 vertebral body length. The caliper was then placed on a metric ruler, and the interval was recorded to the nearest millimeter to obtain more precise measurements for statistical analysis. Care was taken not to measure any distance that had the radiographic opacity of the fat. The SA of heart was measured in the central third region (from the cranial to caudal border of the widest portion of the heart), perpendicular to the LA, and the number of vertebrae was calculated in the same manner as for LA (Figure-1). The LA and SA dimensions of the heart were then added to obtain a vertebrae or heart sum that indicated the heart size relative to body length. The heart size and vertebral length were also determined in millimeters.

**Figure 2:**
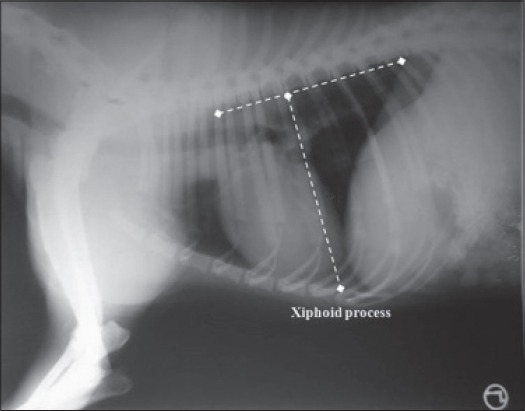
Thoracic depth measured from xiphoid process to the perpendicular of vertebral column in lateral recumbency.

**Figure 3:**
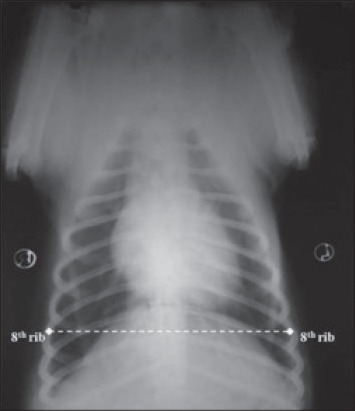
Thoracic width measured as the distance between medial borders of eighth rib at their most lateral curvatures in dorso-ventral recumbency.

Thoracic conformation was determined from the TD to TW ratio, as described by Buchanan and Bucheler [8]. The depth of thorax was measured in the RL radiographic view from the cranial edge of xiphoid process to the ventral border of vertebral column along a line perpendicular to vertebral column (Figure-2). The width of thorax was measured on a dorsoventral radiograph as the distance between medial borders of eighth ribs at their most lateral curvatures (Figure-3). Dogs with a TD to TW ratio of <0.75 were considered to have a broad or barrel thorax, while those with a TD to TW ratio of >1.25 were considered to have a deep thorax. The rest was regarded as having an intermediate chest conformation.

### Statistical analysis

A statistical analysis was performed using SPSS software (SPSS^®^ 17.0 for Windows). Results were expressed as a mean ± standard deviation. Data were analyzed using one-way analysis of variance and Duncan multiple range test for comparison between three breeds. Unpaired Student’s *t*-test and paired Student’s *t*-test were used to compare the differences between male and female dogs and RL versus LL VHS, respectively. One sample *t*-test was used to compare the mean VHS of each breed with Buchanan and Bucheler value (9.7±0.5). Pearson correlation coefficient (r) was calculated to determine the correlation among body weight and TD to TW ratio and VHS. The correlation was considered positive and significant when the correlation coefficient ≥0.40 and significance ≤0.05 (p≤0.05). The significance level for all the tests was p<0.05.

## Results and Discussion

In this study, reference values of VHS in Indian Spitz, Labrador retriever, and Mongrel dogs were established and compared in lateral radiographs. In addition, the effect of certain parameters such as breed, side of radiographic view, gender, body weight, and TD to TW ratio which can affect VHS calculation were determined.

Non-significant differences in the mean age were observed among the dog breeds analyzed. The cardiac measurements of all three dog breeds are summarized in Table-1. VHS in Spitz was similar to Poodles [14] but smaller than Pugs and Pomeranians [12]. VHS in Labrador retriever was greater than Doberman and German shepherd dogs [6] but smaller than Whippets [4], Greyhounds [15], Boxers and Labrador retrievers [10]. VHS in Mongrel dogs was slightly greater than Iranian native, mixed breed [6] and Indian Mongrel dogs [16].

**Table-1:** Mean (±SD) of cardiac measurements in Spitz, Labrador retriever and Mongrel dogs.

Cardiac measurement (v)	Spitz	Labrador retriever	Mongrel	Total
LL LA	5.59±0.27^b^ (5.16.1)	5.55±0.16^b^ (5.35.8)	5.34±0.18^a^ (5.05.6)	5.49±0.24 (5.06.1)
LL SA	4.44±0.20^b^ (4.04.8)	4.67±0.23^c^ (4.35.1)	4.28±0.16^a^ (4.04.5)	4.46±0.25 (4.05.1)
RL LA	5.70±0.25^b^ (5.36.2)	5.69±0.18^b^ (5.45.9)	5.44±0.18^a^ (5.15.8)	5.61±0.24 (5.16.2)
RL SA	4.48±0.23^a^ (4.04.8)	4.71±0.22^b^ (4.35.1)	4.38±0.17^a^ (4.24.7)	4.52±0.24 (4.05.1)
LL VHS	10.03±0.11^b^ (9.910.2)	10.22±0.20^c^ (10.010.6)	9.62±0.25^a^ (9.210.0)	9.96±0.32 (9.210.6)
RL VHS	10.21±0.13b (10.010.4)	10.39±0.19^c^ (10.110.8)	9.82±0.21^a^ (9.410.2)	10.14±0.29 (9.410.8)

Values with different superscripts (a, b, c) differ significantly (p<0.05) between breeds; values with same superscript differ non-significantly (p>0.05). n=Number of animals in each breed, v=Length measured in vertebrae, LL=Left lateral, RL=Right lateral, LA=Long axis, SA=Short axis

In lateral radiographs, VHS differed significantly (p<0.05) among Spitz, Labrador retriever and Mongrel dogs. Labrador retrievers had the highest VHS in LL and RL radiographs (10.22±0.20 v and 10.39±0.19 v, respectively) followed by Spitz (10.03±0.11 v and 10.21±0.13 v, respectively) and Mongrel dogs (9.62±0.25 v and 9.82±0.21 v, respectively). In Mongrel dogs, cardiac LA and SA measurement was significantly (p<0.05) lower than the other two breeds. Similar findings were reported by Ghadiri *et al*. [6] where Doberman dogs had the highest VHS followed by German shepherd and mixed breed dogs while native dogs had the lowest VHS. Such variation in the VHS measurement among different dog breeds could be attributed to the differences in breed thoracic conformation.

LL and RL VHS in Spitz and Labrador retriever was significantly (p<0.001) greater than reference VHS of 9.7±0.5 v. In Mongrel dogs, only RL VHS was significantly (p<0.037) >9.7±0.5 v (Table-2). Values of VHS outside the published reference range have been reported previously in several breeds like Whippets [4], Labrador retriever [7], Boxer, Cavalier King Charles Spaniel and Doberman [10], Pug, Pomeranian, Bulldog and Boston Terriers [12], Poodles [14], Greyhounds [15], Beagle [17], and American Pitbull Terrier [18].

**Table-2:** Comparison of vertebral heart scores of Spitz, Labrador retriever and Mongrel dogs with published reference VHS of 9.7±0.5 vertebrae.

Breed	LL VHS (v)	p value (breed VHS vs. reference)	RL VHS (v)	p value (breed VHS vs. reference)
Spitz	10.03±0.11	<0.0001	10.21±0.23	<0.0001
Labrador retriever	10.22±0.20	<0.0001	10.39±0.19	<0.0001
Mongrel	9.62±0.25	0.242	9.82±0.21	0.037

n=Total number of animal in each breed, LL=Left lateral, RL=Right lateral, VHS=Vertebral heart score, v, length measured in vertebrae

Mean VHS in RL recumbency was significantly (p<0.0001) greater than mean VHS in LL recumbency in all three breeds (Table-3). There are inconsistent views regarding the differences in VHS from radiographs obtained in LL versus RL recumbency in dogs. Some studies ruled out an effect of LL versus RL recumbency on the VHS value in dogs [6-8,15]. While others reported significantly (p<0.05) higher VHS in RL recumbency compared to left [4,6,17] which was similar to the findings reported in our study. Gugjoo *et al*. [7,19] reported that higher VHS in RL recumbency could possibly be due to the fact that greater divergence of X-ray beam coupled with greater distance of heart from the cassette occurs in RL recumbency leading to image magnification. Kraetschmer *et al*. [17] reported that position of heart within the thorax changes slightly as a result of gravity when the animal is restrained in different recumbency leading to change in the VHS. Significant correlation between the measures obtained in LL versus RL recumbency in Spitz (r=0.58; p=0.02), Labrador retriever (r=0.87; p<0.0001), and Mongrel dogs (r=0.93; p<0.0001) (Table-3) was in accordance with the findings in normal dogs [11].

**Table-3:** Comparison and correlation between LL and RL VHS in Spitz, Labrador retriever and Mongrel dogs.

Breed	LL VHS (v)	RL VHS (v)	p value	r	p value for correlation
Spitz	10.03±0.11	10.21±0.23	<0.0001	0.58	0.02
Labrador retriever	10.22±0.20	10.39±0.19	<0.0001	0.87	<0.0001
Mongrel	9.62±0.25	9.82±0.21	<0.0001	0.93	<0.0001
Total	9.96±0.32	10.14±0.29	<0.0001	0.95	<0.0001

n=Total number of animal in each breed, v=Length measured in vertebrae, r=Correlation coefficient, LL=Left lateral, RL=Right lateral, VHS=Vertebral heart score, LL=Left lateral, RL=Right lateral

Non-significant (p>0.05) differences in the VHS between male and female dogs within each breed (Table-4) was in accordance with the findings of Bavegems *et al*.; Gulanber *et al*.; Gugjoo *et al*.; Marin *et al*.;Kraetschmer *et al*. [4,5,7,15,17]. Such non-significant differences in the mean VHS of male and female dogs could be attributed to the fact that there were no distinct differences in the overall body weight and sizes of sexes within each breed, whereas in other canine breeds such differences may be present that might have lead to significant differences in the VHS among sexes [10,12].

**Table-4:** Mean (±SD) of cardiac measurements in male and female dogs within each breed.

Breed	Cardiac measurement (v)	Male (n=10)	Female (n=10)	p value (male vs. female VHS)
Spitz	LL LA	5.58±0.23 (5.46.0)	5.59±0.32 (5.16.1)	0.972
	LL SA	4.45±0.17 (4.24.7)	4.43±0.24 (4.04.8)	0.871
	RL LA	5.69±0.23 (5.56.1)	5.71±0.29 (5.36.2)	0.882
	RL SA	4.47±0.18 (4.24.8)	4.51±0.28 (4.04.8)	0.726
	LL VHS	10.04±0.12 (9.910.4)	10.02±0.10 (9.910.2)	0.826
	RL VHS	10.25±0.09 (10.010.6)	10.16±0.16 (10.110.4)	0.202
Labrador retriever	LL LA	5.52±0.19 (5.35.8)	5.59±0.12 (5.45.7)	0.463
	LL SA	4.74±0.27 (4.35.1)	4.60±0.18 (4.34.9)	0.251
	RL LA	5.65±0.19 (5.45.9)	5.72±0.17 (5.45.9)	0.419
	RL SA	4.77±0.26 (4.35.1)	4.64±0.15 (4.44.9)	0.224
	LL VHS	10.26±0.17 (10.110.6)	10.19±0.23 (10.010.6)	0.483
	RL VHS	10.42±0.19 (10.210.8)	10.36±0.18 (10.110.7)	0.525
Mongrel	LL LA	5.38±0.16 (5.15.6)	5.29±0.20 (5.05.5)	0.297
	LL SA	4.28±0.15 (4.04.5)	4.29±0.17 (4.04.5)	1.000
	RL LA	5.45±0.19 (5.25.8)	5.42±0.19 (5.15.7)	0.798
	RL SA	4.40±0.18 (4.24.7)	4.36±0.16 (4.34.6)	0.671
	LL VHS	9.67±0.20 (9.410.0)	9.57±0.29 (9.210.0)	0.436
	RR VHS	9.85±0.20 (9.610.3)	9.78±0.26 (9.410.2)	0.565

p<0.05, significant. n=Number of animals in each breed, v=Length measured in vertebrae, LL=Left lateral, RL=Right lateral, LA=Long axis, SA=Short axis, SD=Standard deviation

To know the type of thoracic conformation of three dog breeds, TD to TW ratio was calculated. TD as well as TW differed significantly (p<0.05) among Spitz, Labrador retriever, and Mongrel dogs while TD to TW ratio did not differ significantly between the sexes and among the dog breeds (Table-5). A correlation analysis between VHS and TD to TW ratio was performed to determine whether chest depth was responsible for variation in VHS among dog breeds. There was no significant correlation between the type of chest and VHS in all dog breeds (Table-6). TD to TW ratio of all dogs ranged 0.75-1.25, suggestive of intermediate chest conformation. None of the dogs had wide or deep chest conformation, which could be a reason for the lack of significant correlation between VHS and type of chest in our study. Similar findings were observed by Jepsen-Grant *et al*. [12] in Pug, Pomeranian, Yorkshire terrier, Daschund, Bulldog, Shih Tzu, Lhasa Apso and Boston Terriers, Castro *et al.*, [13] in Yorkshire Terriers and Basile [20] in British Bulldog. Non-significant effect of gender, size of dog and thoracic conformation on the VHS in normal dogs was reported by Greco *et al*. [11].

**Table-5:** TD, TW and TD to TW ratio in Spitz, Labrador retriever and Mongrel dogs.

Cardiac measurements	Gender	Spitz	Labrador retriever	Mongrel
TD	M	10.82±1.39	18.17±1.82	16.48±1.06
	F	10.90±1.44	17.22±2.17	14.29±1.06
	T	10.86±1.37^a^	17.70±2.00^c^	15.38±1.53^b^
TW	M	13.15±1.11	20.34±1.39	17.62±1.09
	F	13.28±1.45	19.04±2.36	15.56±1.08
	T	13.21±1.28^a^	19.68±1.99^c^	16.59±1.49^b^
TD to TW ratio	M	0.82±0.06	0.90±0.03	0.93±0.01
	F	0.79±0.06	0.89±0.06	0.92±0.01
	T	0.82±0.05^b^	0.89±0.04^b^	0.93±0.01^b^

Values with different superscripts (a, b, c) differ significantly between breed groups (p<0.05); values with same superscript differ nonsignificantly (p>0.05). n=Number of animals, TD=Thoracic depth, TW=Thoracic width, M=Male, F=Female, T=Total

**Table-6:** Pearson correlation coefficients of LL and RL VHS to TD to TW ratio and body weight in Spitz, Labrador retriever and Mongrel dogs.

Cardiac parameters	Correlation coefficient (r)			

	Spitz	Labrador retriever	Mongrel	p value for correlation
LLVHS and TD:TW	−0.067	−0.31	−0.026	NS
RLVHS and TD:TW	−0.215	−0.27	0.06	NS
LL VHS and body weight	−0.077	0.034	0.383	NS
RL VHS and body weight	0.076	0.054	0.387	NS

LL=Left lateral, RL=Right lateral, TD=Thoracic depth, TW=Thoracic width, VHS=Vertebral heart score, R=Correlation coefficient, NS=Nonsignificant

Body weight did not correlated significantly (p>0.05) with VHS in Spitz, Labrador retriever and Mongrel dogs (Table-6). A similar finding was reported by Basile [20] in English Bulldogs where body weight had no influence on the VHS within any of the radiographic projections. Contrary to this, significant correlation between the VHS and body weight on lateral and ventrodorsal radiographs was observed by Castro *et al*. [13] in Yorkshire Terriers having a homogeneous sample weight and age.

Mongrel dogs in our country are commonly used as companion animals in large numbers. Unlike other dog breeds, they are not registered and therefore, there is no reference range for their characteristic. These dogs have an intermediate chest conformation, just like Spitz and Labrador retrievers. As Mongrel dogs had a smaller mean VHS than other two breeds, their heart sizes were on average, slightly smaller than the other two breeds included in our study.

## Conclusion

The breed and recumbent side of radiographic view should be taken into consideration while calculating VHS in dogs to avoid any erroneous interpretation of cardiac enlargement.

## Authors’ Contributions

DB: Research was done by this author as part of her PhD thesis dissertation. MH and ACS: Designed the study and supervised the research. MBG: Provided valuable suggestions regarding the design of research and analysis of data collected during research. DB and JKC: Worked and collaborated in the experiment and compilation of the results as well as the manuscript. All authors read and approved the final manuscript.
